# Consecutive Dual-Session Transcranial Direct Current Stimulation in Chronic Subjective Severe to Catastrophic Tinnitus with Normal Hearing

**DOI:** 10.3390/jpm14060577

**Published:** 2024-05-28

**Authors:** Sung Jun Han, Ji Hye Lee, Yeso Choi, Seok Min Hong, Jun Hee Kim, Sung Kyun Kim

**Affiliations:** 1 Department of Otolaryngology-Head & Neck Surgery, Hallym University College of Medicine, Chuncheon 24253, Republic of Korea; 2 Department of Otolaryngology-Head & Neck Surgery, Kyung Hee University College of Medicine, Seoul 02447, Republic of Korea; 3 Department of Otolaryngology-Head & Neck Surgery, Kresge Hearing Research Institute, University of Michigan Medical School, Ann Arbor, MI 48105, USA; 4 Laboratory of Brain & Cognitive Sciences for Convergence Medicine, Hallym University College of Medicine, Anyang 14068, Republic of Korea

**Keywords:** transcranial direct current stimulation, tinnitus, chronic, subjective, treatment

## Abstract

Transcranial direct current stimulation (tDCS) is emerging as a promising non-invasive intervention for tinnitus by aiming to modulate abnormal brain activity. This study investigated the efficacy of dual-session tDCS for the relief of perception, distress, and loudness in patients with severe chronic subjective tinnitus and assessed the duration of tinnitus suppression effects compared to single-session and control groups over a 2-month follow-up. In a prospective, randomized, single-blind, placebo-controlled trial, 30 participants with severe chronic subjective tinnitus underwent bifrontal tDCS. The control group (n = 9), single-session group (n = 10), and dual-session group (n = 11) received 2 mA stimulation for 20 min per session, twice a week for one month. The treatment response was monitored weekly using the Visual Analogue Scale (VAS), with additional assessments using the Tinnitus Handicap Inventory (THI) and Beck Depression Inventory (BDI) at the fourth and eighth weeks. The single- and dual-session groups showed statistically significant improvements in VAS, THI, and BDI scores compared to the control group. THI and BDI scores showed a significant difference between the single- and dual-session groups. The dual-session group demonstrated a more sustained tinnitus suppression effect than the single-session group. tDCS has been validated as an effective intervention for the suppression of tinnitus, with the dual-session protocol showing longer-term benefits. These findings support the potential of tDCS as a treatment for tinnitus, particularly in dual-session applications.

## 1. Introduction

Tinnitus is defined as the perception of sound in the absence of an external source. Approximately 15–30% of the global population is affected by tinnitus in their lives in the United States, and the prevalence of tinnitus is reported to be increasing [1]. The prevalence of tinnitus in South Korea is estimated to be 238 per 1000 people and increases with age, especially after middle age [2]. Tinnitus can have a negative impact on an individual’s overall quality of life, like sleep, mood, and daily performance [3]. Although there are various auditory functional mechanisms emerging as the cause of tinnitus, it remains controversial [4,5,6]. For this reason, many treatment modalities such as medication treatment, psychological support, and symptomatic treatment have been tried in clinical and preclinical studies, but they are not curative.

Increased burst activity of the dorsal cochlear nucleus (DCN) cells in the auditory brainstem has been considered one of the mechanisms of tinnitus [7,8]. DCN hyperactivity can be associated with tinnitus in various types of hearing loss, including noise-induced hearing loss [9]. However, tinnitus can develop in a clinically normal hearing population that shows normal findings in conventional audiologic tests. Tinnitus in normal hearing can be explained by cochlear synaptopathy, an electrophysiological dysfunction of the ribbon synapses in the inner ear. Furthermore, central disinhibition in the brain circuits that process auditory signals can be evidence of tinnitus in those with normal hearing [10]. It has also been reported that involvement of the limbic system, which is responsible for processing somatic sensation, emotion, and memory, can lead to anxiety and depressive mood and further aggravate tinnitus [11,12].

Transcranial direct current stimulation (tDCS) is one of the relatively new treatment options as a non-invasive method that can modulate abnormal activity in the brain to alleviate tinnitus symptoms. tDCS initially attracted attention for the purpose of modulating the activity of specific brain regions implicated in cognitive, motor, and affective disorders [13]. The targets to be stimulated through tDCS in tinnitus are mainly the auditory cortex [14,15], dorsolateral prefrontal cortex (DLPFC) [16,17], and left temporoparietal area (LTPA) [18], which are different for each study. Most studies reported significant improvement in patients who have tinnitus with tDCS treatment, but the duration of tinnitus suppression varies from minutes to several days [19,20,21]. Additionally, studies to date have conducted tDCS on chronic subjective tinnitus, but the subjects’ loudness, distress of tinnitus, level of hearing threshold, and psychiatric comorbidity have varied, and there is no report on whether there is an effect of suppressing tinnitus through stimulation several times a day. Furthermore, there are few studies focusing on the feasibility of tDCS only for severe to catastrophic chronic subjective tinnitus.

Here, we aimed to analyze the effect of continuous dual-session tDCS in patients with severe chronic subjective tinnitus without any evidence of hearing loss in terms of relief of tinnitus perception, distress, and loudness. Also, we investigated the difference in the maintenance duration of the tinnitus suppression effect of consecutive dual-session tDCS compared to other groups followed up for 2 months.

## 2. Materials and Methods

### 2.1. Study Design and Ethics Statement

This prospective, randomized, single-blind, placebo-controlled trial was conducted at the Department of Otolaryngology, Hallym University Dongtan Sacred Heart Hospital, Hwaseong, South Korea. This clinical trial has been registered at ClinicalTiral.gov (ID: NCT06423742). The study was conducted according to the guidelines of the Declaration of Helsinki, and the Institutional Review Board of Hallym University College of Medicine (IRB# 2021-11-009) permitted this study. Written informed consent was obtained from all subjects involved in the study. All analyses adhered to the guidelines and regulations of the ethics committee of Hallym University.

### 2.2. Participants

We enrolled individuals who visited the out-patient clinic as new patients presenting with tinnitus from 15 December 2021 to 30 September 2022. Subjects included were individuals over 18 years of age who had suffered from subjective tinnitus for over 3 months. Also, we evaluated the results of pure tone audiometry (PTA), speech audiometry (SA), and auditory brainstem response (ABR). Participants were considered to have normal hearing when the average pure tone threshold between 0.5–4 KHz was less than 25 dB, the V wave was shown at 30 dBnHL, and the difference in V wave latency of 90 dBnHL in the ABR test was less than 0.2 ms in both ears. Also, we enrolled participants who have severe tinnitus with a Tinnitus Handicap Inventory (THI) score of 58 or higher and whose symptoms of anxiety and depressive mood have been assessed through the Beck Depression Inventory (BDI) questionnaire.

Subjects were excluded if they needed to be treated for neurological disorders such as epilepsy, migraine, or intracranial mass. We also excluded participants who were pregnant on the day of agreeing to consent and who have metal-based electric implantable prosthetics. Furthermore, candidates were excluded with the following: objective or somatic tinnitus, auditory hallucinations, unwillingness to agree to the written consent or continue the trial, or participation in previous trials with tDCS for tinnitus.

### 2.3. Trial Design

**Figure 1:**
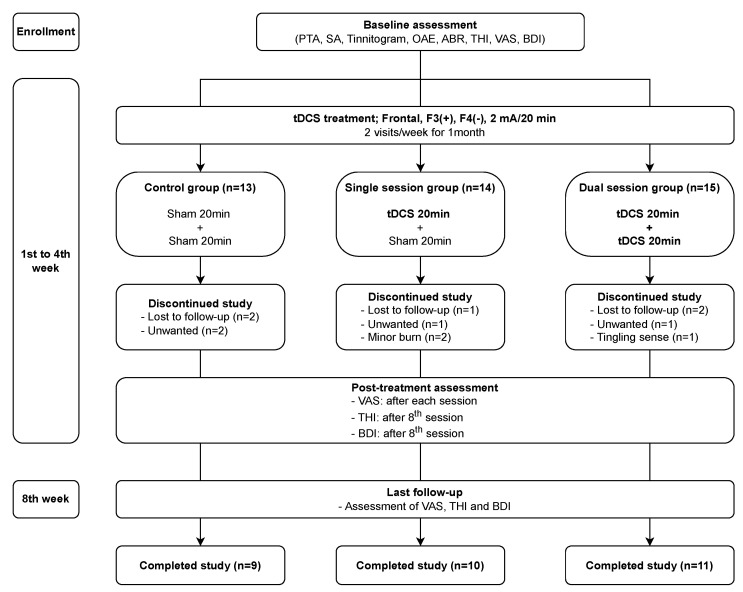
Schematic flow of the study. Abbreviations: PTA = pure tone audiometry; SA = speech audiometry; OAE = otoacoustic emission; ABR = auditory brain stem response; THI = Tinnitus Handicap Inventory, BDI = Beck Depression Inventory; tDCS = transcranial direct current stimulation.

This study was aiming to evaluate the therapeutic effect of consecutive dual-session tDCS compared with sham and single-session groups. The participants were randomly allocated to three different arms (control, single session, and dual session) using blocks to balance the size of each group. Pure tone audiometry; speech audiometry; tinnitogram (pitch matching, loudness, minimal masking level, and residual inhibition); auditory evoked potential; THI; Visual Analogue Scale (VAS) of loudness, awareness, and annoyance; and BDI were evaluated as a baseline tests. Participants who were assigned to the control group underwent two sham stimulations per day twice a week for 1 month. Patients received sham and true stimulations, once each, alternately in the single-session group and received two true stimulations per day in the dual-session group for the same period as the control group. All subjects who enrolled in this study were given conventional treatment such as tinnitus retraining therapy, sound therapy using a sound generator, and medications like clonazepam and selective serotonin reuptake inhibitors for patients who wanted relief of symptoms (Figure 1).

### 2.4. Intervention

The subjects in the control group received two consecutive sessions of sham stimulation twice a week for 4 weeks (8 sessions in total). Patients enrolled in the single-session group received both sham stimulation and active tDCS in random order during two consecutive sessions. In the dual-session group, the patients underwent two consecutive tDCS sessions. All patients were given directive counselling like tinnitus rehabilitation therapy (TRT) and sound therapy with a sound generator as a conventional treatment. Clonazepam was prescribed to seven patients in the control group who wanted to take the medication. None of the 20 patients enrolled in the tDCS group were prescribed clonazepam.

The device used for tDCS in this study was a DC-Stimulator Plus (NeuroConn GmbH, Ilmenau, Germany). The localization of the stimulation area (DLPFC) was determined according to the 10/20 EEG system. A non-conducting, elastic head strap was placed around the head to prevent displacement during stimulation. The recording electrode covered with a 5 × 7 cm size traditional rectangular sponge was soaked with 6–7 mL of 0.9% NaCl saline solution per side and placed on the F3 (anode, left frontal) and F4 (cathode, right frontal) EEG locations. Subjects were given 2 mA stimulation intensity for 20 min per session, including fade-in and fade-out times for 20 s each. The interval between each session was 20 min. The sham procedure adopted a fade-in of current, short stimulation, fade-out (FISSFO) protocol that consists of an initial ramp-up for 20 s, 2 mA stimulation for 40 s, and 20 s for ramp-down. To check the impendence, a brief current of 110 µA over 15 ms every 550 ms was delivered.

### 2.5. Follow-up and Assessment of Treatment Response

All participants were checked with the VAS immediately after the last stimulation every week for 4 weeks and after the 8th week (4 weeks after the end of treatment). The THI and BDI were assessed at the 4th and 8th week visits. It has been suggested that an improvement in each subscale of the VAS score of ≥2 points and THI score ≥ 20 can be indicated as a good response. We also considered a good response as a BDI score of less than 10 after treatment. A study flow diagram is shown in Figure 1.

### 2.6. Statistical Analyses

A Pearson’s Chi-square test was used for comparing sex and laterality. A one-way ANOVA was used for comparing age, initial THI, initial BDI, and VAS—Loudness (VAS-Lo). A Kruskal–Wallis test was used to compare initial threshold of PTA, VAS—Annoyance (VAS-An), and VAS—Awareness (VAS-Aw). A repeated measures ANOVA was used to assess the treatment outcomes. A paired T-test and Wilcoxon signed-rank test were used to compare changes in VAS scores, THI, and BDI before and after treatment at each point. Responder rates were examined using the Fisher’s exact test. All tests were performed in SPSS v.28 and *p* < 0.05 was considered as significant.

## 3. Results

### 3.1. Demographic Data of Patients

The demographic data of the 30 patients who completed the study are shown in Table 1. There was no significant difference in the age, sex, threshold of PTA, initial THI, VAS score, and BDI among the three groups. Twenty-one patients were considered to have borderline clinical depression (score of 17–20) or moderate depression (score of 21–30) based on their BDI score.

### 3.2. Change in VAS, THI, and BDI Score after Treatment

**Figure 2:**
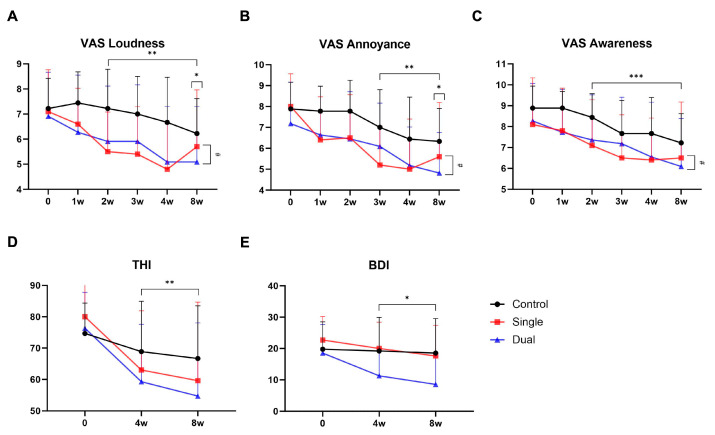
Change in VAS, THI, and BDI score after treatment. (**A**) The control group showed a statistically significant tinnitus suppression effect at the 8th week (*), while both the single-session group and the dual-session group showed significant effects starting from the 2nd week (**). (**B**) Similar to the loudness scores, the control group showed therapeutic effects in the awareness score at the 8th week (*), while both the single-session group and the dual-session group showed significant effects starting from the 3rd week (**). (**C**) The awareness score showed a significant decrease in all three groups starting from the 2nd week (***). Single-session group exhibited a rebound in VAS scores at the 8th week, whereas the dual-session group showed sustained tinnitus suppression effects throughout the observation period (#). (**D**) Both single- and dual-session groups demonstrated statistically significant improvements in THI at the fourth and eighth week (**). (**E**) The dual-session group was the only group that showed improvement in BDI score compared with control group (*). * *p* < 0.05, ** *p* < 0.01, *** *p* < 0.001. Abbreviations: VAS = Visual Analogue Scale; THI = Tinnitus Handicap inventory; BDI = Beck Depression Inventory.

At the 8-week mark, the control group exhibited a significant reduction (*p* = 0.024) in tinnitus loudness compared to initial scores. Both the single- and dual-session groups displayed significant improvements from the second week post-treatment, with their scores from the first to the eighth week significantly better than those of the control group. However, no statistical difference was observed between the single- and dual-session groups (Figure 2A). Like the loudness scores, the control group’s annoyance scores significantly decreased at the 8-week follow-up (*p* = 0.007). The dual-session group showed significant improvements at all measured time points, starting at the third week. No differences were found between the single- and dual-session groups (Figure 2B). Improvements in tinnitus awareness were significant from the second week across all groups. As with loudness and annoyance, no significant differences were noted between the single- and dual-session groups (Figure 2C). Notably, while no significant differences were recorded, the 8-week subscale changes suggested a slight uptick in the single-session group from the fourth week, whereas the dual-session group continued to improve. For the THI score, no significant changes were observed in the control group at any time point. In contrast, both single- and dual-session groups demonstrated statistically significant improvements at the fourth (*p* = 0.012 in the single-session group; *p* = 0.007 in the dual-session group) and eighth weeks (*p* = 0.011 in the single-session group; *p* = 0.008 in the dual-session group) compared to the initial assessment, with no notable differences in relation to the number of tDCS sessions (Figure 2D). At the fourth- and eighth-week marks, the BDI scores of the control and single-session groups did not show significant changes from baseline. The dual-session group, however, exhibited significant improvements at both time points, with a notably greater enhancement compared to the single-session group (*p* = 0.005, 0.003 at each time point) (Figure 2E).

### 3.3. Responder Rate

**Figure 3:**
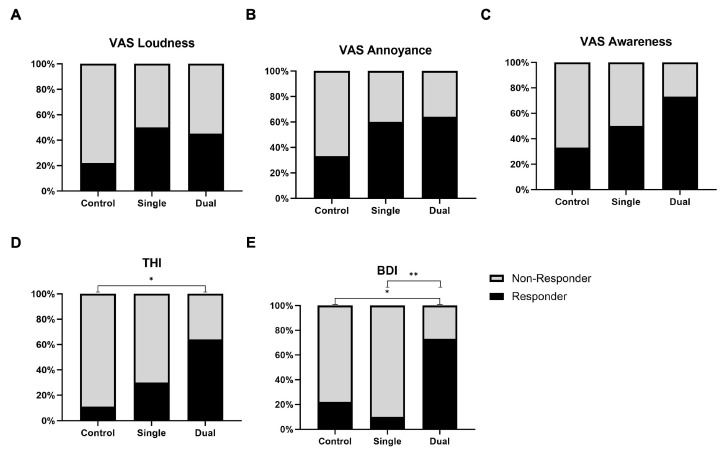
The responder rate in VAS, THI, and BDI score after treatment. It has been suggested that when an improvement in each subscale of VAS score of ≥2 points and THI score ≥20 can be indicated as a good response. (**A**–**C**) In all VAS scores, both the single-session group and the dual-session group demonstrated better treatment effects compared to the control group, although there was no statistically significant difference. (**D**) When comparing the dual-session group to the control group, a statistically significant difference was observed in the responder rate for THI scores (*). (**E**) The responder rate for BDI scores in the dual-session group was significantly different to that of the control (*) and single-session groups (**). * *p* < 0.05, ** *p* < 0.01. Abbreviations: VAS = Visual Analogue Scale; THI = Tinnitus Handicap inventory; BDI = Beck Depression Inventory.

For the improvement of VAS loudness score, both single- (50.0%) and dual-session (45.5%) groups showed a significant difference (*p* = 0.008 in single session, *p* = 0.011 in dual session) compared to the control group (22.2%). There was no statistical difference in responder rates between the single- and dual-session groups (Figure 3A). The responder rate for the VAS annoyance score was significantly different in the treatment groups (single: 60.0%, *p* = 0.013; dual: 63.3%, *p* = 0.021) compared to the control group (33.3%). There was no distinction based on the number of treatments (Figure 3B). In the VAS awareness score, 33.3% of the control group responded to treatment, with a significantly higher response rate in both the single-session (50.0%, *p* = 0.004) and dual-session (72.73%, *p* = 0.001) groups (Figure 3C). A comparison of THI scores before and after treatment revealed that the dual-session group (63.6%) had a statistically significant difference in responder rate compared to the control group (*p* = 0.028) (Figure 3D). The responder rate for BDI scores in the dual-session group (72.7%) was significantly superior to that of the control (22.2%) and single-session groups (10.0%) (*p* = 0.035 and *p* = 0.008) (Figure 3E).

## 4. Discussion

The prevalence of tinnitus and its detrimental effects on psychiatric health have become increasingly prominent in medical research, with many patients seeking resolution for this persistent condition in otology clinics. The strong correlation between tinnitus and psychiatric disorders, including its potential links to suicidality, heightens the need for effective interventions [22]. Our study adds to the growing evidence that tDCS, as a non-invasive neuromodulatory intervention, may offer symptomatic relief for tinnitus sufferers.

tDCS is widely used alongside repetitive transcranial magnetic stimulation (rTMS) as a non-invasive neuromodulation treatment for both acute and chronic tinnitus. However, the effectiveness of tDCS on severe to catastrophic levels of chronic subjective tinnitus, as we focused on in our study, has not been previously reported. This gap in the literature is significant given the substantial mental health burden associated with severe to catastrophic tinnitus.

In the current climate, where telemedicine and at-home treatments are becoming the norm, tDCS stands out for its user-friendly application. This characteristic is especially beneficial in an aging society, where it offers fewer side effects and is suitable even for patients with limited mobility. While repetitive transcranial magnetic stimulation (rTMS) is known for its effectiveness in various psychiatric disorders and short-term tinnitus relief, its long-term effects remain unverified. tDCS, akin to rTMS, has been established as effective for short-term relief [19]. Our study focuses on whether repeated sessions could enhance the duration of its therapeutic effects.

This study demonstrated the effectiveness of not only single-session tDCS but also dual-session tDCS compared to the control group. We observed a significant decrease in nearly all tinnitus indices in both the single-session and dual-session groups. Depressive mood is a commonly associated comorbidity in severe to catastrophic chronic subjective tinnitus, and this relationship is very close [23]. Since tinnitus is challenging to evaluate objectively, a questionnaire-based assessment tool is used to measure functional disability and estimate a psychological state such as mood and anxiety resulting from tinnitus. Hence, when assessing the impact of neuromodulation on tinnitus, the interpretation may be ambiguous, as it could pertain to its effect on depressive mood or its effect on tinnitus itself. Importantly, our research results demonstrated a significant decrease not only in these subjective indicators of distress but also in the perceived loudness subjective indicator.

We observed that the VAS loudness scores indicated an earlier tinnitus suppression effect in the treatment groups compared to the control group, with the single-session group showing earlier improvement than the dual-session group. All groups exhibited significant suppression in VAS awareness scores from the second week. There was no change in the THI scores in the control group, while the treatment groups showed significant improvement from the eighth week, supporting the multifactorial nature of tinnitus that involves a complex interplay of various brain regions.

Our study was conducted on patients with severe to catastrophic tinnitus, and 70% of these patients exhibited comorbid borderline to moderate depression. Ongoing research findings consistently report that tDCS can reduce depressive symptoms in major depressive disorders and other psychiatric conditions [24,25]. While it remains uncertain whether the significant improvement in the BDI is a secondary reduction due to tDCS-induced tinnitus suppression or a direct impact of tDCS on emotions, we have confirmed a noteworthy improvement in depressive symptoms. This reaffirms the psychiatric efficacy of tDCS, suggesting the potential for tDCS to be a remarkable treatment approach for patients suffering from both tinnitus and depression simultaneously.

After treatment with tDCS, all VAS scores decreased significantly in both groups, but we found slightly different characteristics between the two groups. The final VAS scores in both groups somewhat decreased compared to before treatment. Interestingly, the dual-session group exhibited a continuous decrease in tinnitus symptoms throughout the entire follow-up period. In contrast, the single-session group initially showed a reduction in tinnitus symptoms until the fourth week, and the final VAS score showed a slight increase. While the exact duration of the treatment effect of tDCS remains unclear, the potential for a long-term tinnitus suppression effect was observed in the dual-session group, emphasizing the necessity for further research with longer follow-up periods. In addition to controlling the number of sessions per day, tDCS has many adjustable factors such as stimulation intensity, electrode location, and stimulation duration [26]. Indeed, it will be possible to shed light on the tinnitus suppression effect of tDCS through detailed research that appropriately adjusts these factors.

A major strength of our study is the rigorous methodological approach, including a randomized, single-blind, placebo-controlled trial design, which strengthens the validity of our findings. Additionally, our focus on both single and dual-session tDCS provides valuable insights into the optimization of treatment protocols.

However, our study is not without limitations. The sample size is relatively small, which may limit the generalizability of our results. The single-blind design might have introduced some bias, as participants could potentially infer their group assignment. Moreover, the duration of the follow-up period, although informative, may not capture the full long-term effects of the treatment. Future studies with larger sample sizes and double-blind methodologies are necessary to confirm and extend our findings.

## 5. Conclusions

In sum, our research bolsters the body of evidence endorsing tDCS as a promising intervention for tinnitus, with the dual-session regimen showing potential for sustained symptom management. Future research, with larger sample sizes and double-blind protocols, is needed to refine tDCS treatment parameters, thereby solidifying its place as a cornerstone in the therapeutic landscape for tinnitus.

## Figures and Tables

**Table 1:** Demographic characteristics of the patients.

	Control (n = 9)	Single Session (n = 10)	Dual Session (n = 11)	*p* Value
Age (years)	47.14 ± 9.14	46.62 ± 12.03	44.11 ± 9.31	0.776
Sex				
Male	4	4	5	
Female	5	6	6	
Laterality				
Unilateral	6	7	7	
Bilateral	3	3	4	
PTA(R)	15.74 ± 7.86	13.92 ± 4.57	12.12 ± 7.01	0.482
PTA(L)	14.44 ± 7.86	17.50 ± 9.91	12.73 ± 7.03	0.429
Initial THI	74.67 ± 9.70	80.00 ± 14.58	76.36 ± 11.41	0.619
VAS-Lo	7.22 ± 1.20	7.10 ± 1.66	6.90 ± 1.76	0.905
VAS-An	7.88 ± 1.27	8.0 ± 1.56	7.18 ± 1.99	0.482
VAS-Aw	9.0 ± 1.12	8.10 ± 2.23	8.27 ± 1.79	0.523
BDI	19.77 ± 8.74	22.7 ± 7.48	18.45 ± 9.13	0.635

Abbreviations: PTA = pure tone audiometry; THI = Tinnitus Handicap Inventory; VAS-Lo = Visual Analogue Scale—Loudness; VAS-An = Visual Analogue Scale—Annoyance; VAS-Aw = Visual Analogue Scale—Awareness; BDI = Beck Depression Inventory.

## Data Availability

The raw data supporting the conclusions of this article will be made available by the authors on request.
